# (3a*R*,6a*R*)-1-Phenyl-5-[(*R*)-1-phenyl­ethyl]-3-[4-(trifluoro­meth­yl)phen­yl]-1,6a-dihydro­pyrrolo[3,4-*c*]pyrazole-4,6(3a*H*,5*H*)-dione

**DOI:** 10.1107/S1600536809049319

**Published:** 2009-11-25

**Authors:** Chris F. Fronczek, Yaşar Dürüst, Muhammet Yildirim, Frank R. Fronczek

**Affiliations:** a Department of Chemistry, Louisiana State University, Baton Rouge, LA 70803-1804, USA; b Department of Chemistry, Abant Izzet Baysal University, TR-14280 Bolu, Turkey

## Abstract

In the title mol­ecule, C_26_H_20_F_3_N_3_O_2_, the two central five-membered rings form a dihedral angle of 62.94 (8)°. The absolute configuration was determined by analysis of Bijvoet pairs based on resonant scattering of light atoms, yielding a Hooft parameter *y* = −0.05 (11). Notable intra- and inter­molecular contacts include C—H⋯O and C—H⋯π(arene) hydrogen bonds.

## Related literature

For cyclo­addition reactions of chiral maleimides with dipolar compounds, see: Bienayme (1997 ▶); Blanarikova *et al.* (2001 ▶); Chihab-Eddine *et al.* (2001 ▶); Oishi *et al.* (1993 ▶, 1999 ▶, 2007 ▶); Ondrus & Fisera (1997 ▶); Tokioka *et al.* (1997 ▶). For the determination of the absolute configuration by Bayesian analysis of Bijvoet differences, see: Hooft *et al.* (2008 ▶). For a description of the Cambridge Structural Database, see: Allen (2002 ▶). For related structures, see: Hursthouse *et al.* (2003 ▶); Skof *et al.* (1998 ▶); Fronczek *et al.* (2009 ▶).
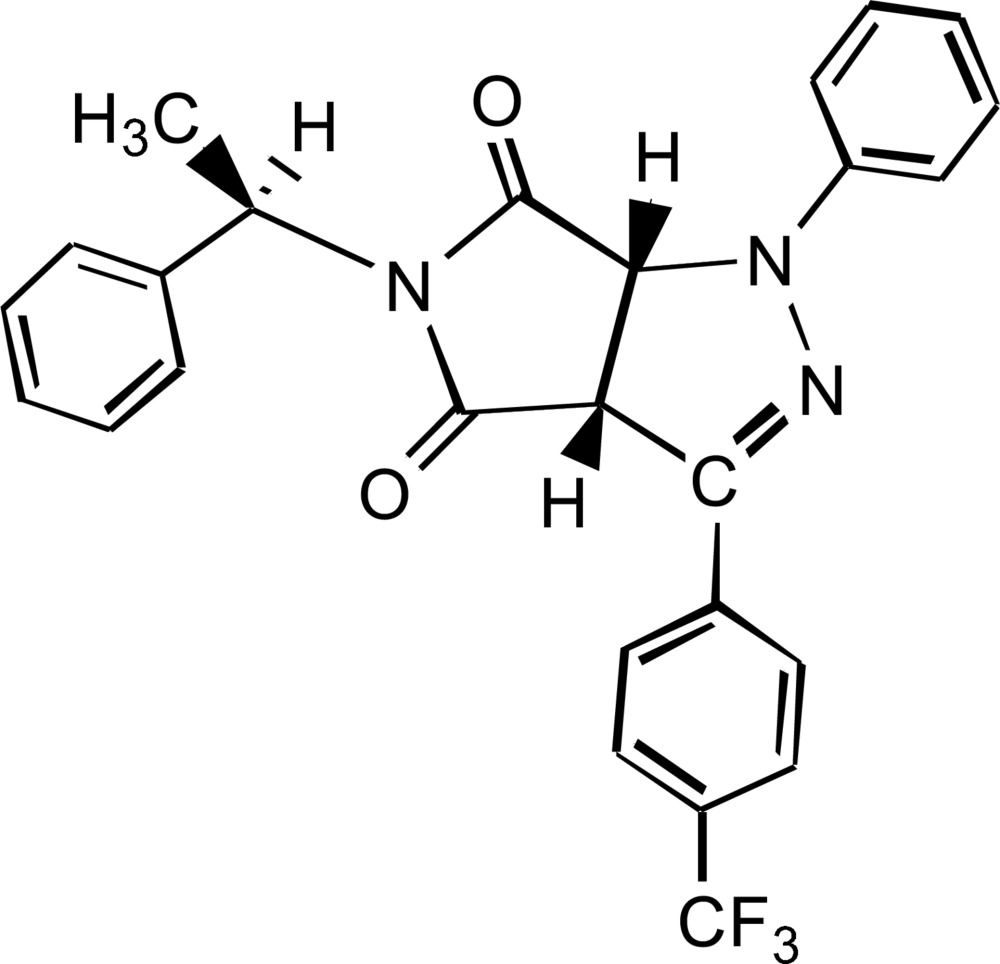


## Experimental

### 

#### Crystal data

C_26_H_20_F_3_N_3_O_2_
*M* *_r_* = 463.45
Orthorhombic, 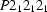

*a* = 8.7982 (15) Å
*b* = 9.3064 (15) Å
*c* = 25.992 (4) Å
*V* = 2128.2 (6) Å^3^
*Z* = 4
Cu *K*α radiation
μ = 0.93 mm^−1^
*T* = 90 K
0.30 × 0.18 × 0.03 mm

#### Data collection

Bruker Kappa APEXII CCD area-detector diffractometer
Absorption correction: multi-scan (*SADABS*; Sheldrick, 2004 ▶) *T*_min_ = 0.767, *T*_max_ = 0.972
20719 measured reflections
3822 independent reflections
2959 reflections with *I* > 2σ(*I*)
*R*_int_ = 0.069

#### Refinement

*R*[*F* ^2^ > 2σ(*F* ^2^)] = 0.043
*wR*(*F* ^2^) = 0.108
*S* = 1.03
3822 reflections
309 parameters
H-atom parameters constrained
Δρ_max_ = 0.31 e Å^−3^
Δρ_min_ = −0.18 e Å^−3^
Absolute structure: Flack (1983 ▶), 1590 Friedel pairs
Flack parameter: −0.2 (2)

### 

Data collection: *APEX2* (Bruker, 2006 ▶); cell refinement: *SAINT* (Bruker, 2006 ▶); data reduction: *SAINT*; program(s) used to solve structure: *SHELXS97* (Sheldrick, 2008 ▶); program(s) used to refine structure: *SHELXL97* (Sheldrick, 2008 ▶); molecular graphics: *ORTEP-3 for Windows* (Farrugia, 1997 ▶); software used to prepare material for publication: *SHELXTL* (Sheldrick, 2008 ▶).

## Supplementary Material

Crystal structure: contains datablocks global, I. DOI: 10.1107/S1600536809049319/bx2247sup1.cif

Structure factors: contains datablocks I. DOI: 10.1107/S1600536809049319/bx2247Isup2.hkl

Additional supplementary materials:  crystallographic information; 3D view; checkCIF report

## Figures and Tables

**Table 1:** Hydrogen-bond geometry (Å, °)

*D*—H⋯*A*	*D*—H	H⋯*A*	*D*⋯*A*	*D*—H⋯*A*
C18—H18⋯O1	1.00	2.47	2.859 (4)	103
C8—H8⋯*Cg*1^i^	0.95	2.58	3.491 (3)	161

Symmetry code: (i) 
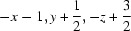
. *Cg*1 is the centroid of the C19–C24 ring.

**Fig. 1.**
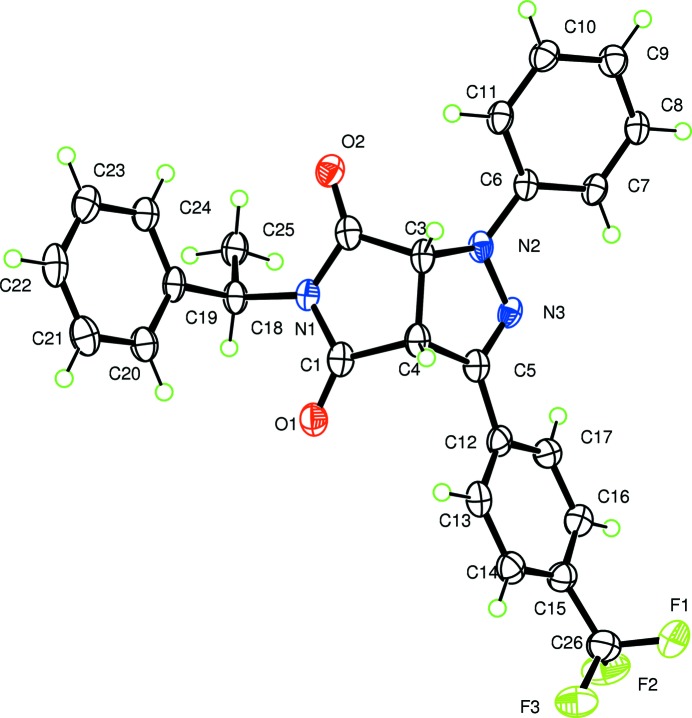
The molecular structure of compound (I), showing the atom-labeling scheme. Displacement ellipsoids are drawn at the 50% probability level, with H atoms having arbitrary radius.

**Fig. 2.**
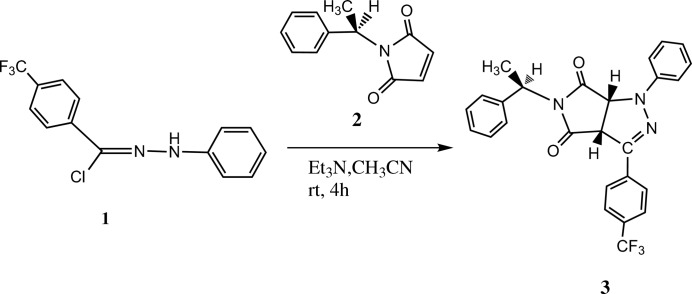
The formation of the title compound.

## supplementary crystallographic information

### Comment

There are few examples of cycloaddition reactions of chiral maleimides with dipolar compounds like nitrones, nitriloxides and anthrones reported in the literature (Bienayme, 1997; Blanarikova *et al.*, 2001; Chihab-Eddine *et al.*, 2001; Oishi *et al.*, 1993; 1999; 2007; Ondrus & Fisera, 1997; Tokioka *et al.*, 1997). Apparently, the only previously-reported example of 1,3-dipolar cycloaddition of C,*N*-substituted nitrilimines to chiral maleimide, (*R*)—*N*-(1-phenylethyl) maleimide is our previous work (Fronczek *et al.*, 2009). Herein, we report the synthesis, characterization and crystal structure of the diastereomer obtained from the above reaction.

The two 5-membered rings at the core of this molecule are essentially planar and form a dihedral angle of 62.94 (8)°. The mean deviation of the seven pyrrolidine-2,5-dione atoms from their least-squares plane is 0.021 Å, and the mean deviation for the 4,5-dihydro-1*H*-pyrazole ring is 0.013 Å. Atom N2 deviates most from the 4,5-dihydro-1*H*-pyrazole ring, with deviation 0.0198 (17) Å. Atom N1 deviates most from the pyrrolidine-2,5-dione ring, with deviation 0.0610 (19) Å. The core of this structure is nearly identical to that found in a recently-reported compound produced in a similar reaction (Fronczek *et al.*, 2009), except that it was the diastereomer with N2 and C5 swapped, and *p*-acetate substituent on phenyl rather than CF_3_. That compound had dihedral angle between the two 5-membered rings 63.66 (4)°. Similar results can also be found in compounds having refcodes CIRFEP and WIQBIH from the Cambridge Structural Database (Allen, 2002, version 5.30, Nov. 2008). In CIRFEP (Hursthouse *et al.*, 2003), the dihedral angle between the central ring planes is 63.65 (9)°, for one of two independent molecules and 64.23 (9)° for the other. For WIQBIH, (Skof *et al.*, 1998), the dihedral angle formed by the central ring planes 65.99 (6)°. Notable intra and intermolecular contacts include C—H···O and C—H···π(arene) hydrogen bonds, Table 1.

The absolute configuration, based on resonant scattering of the light atoms, was slightly ambiguous from of the Flack (1983) parameter, *x* = -0.2 (2). Analysis of the Bijvoet pairs using the method of Hooft *et al.* (2008) yielded a more decisive *y* = -0.10 (7), corresponding to a probability P2(true) = 1.000 for this structure, confirming the absolute configuration. It agrees with that of the starting materials.

### Experimental

*C*-(4-Trifluoromethyl)-*N*-phenyl hydrazonyl chloride 1 (0,149 g, 0.5 mmol) and (*R*)—*N*-(1-phenylethyl) maleimide 2 (0,100 g, 0.5 mmol) were dissolved in dry acetonitrile (20 ml). Et_3_N (0.404 g, 4 mmol) was added dropwise into the mixture with stirring and after addition was completed, the reaction mixture was stirred at room temperature for 2 h. The progress of the reaction was monitored by TLC. When the starting materials disappeared, the solvent, CH_3_CN was evaporated under the reduced pressure and the crude reaction mixture was taken into water (50 ml) to remove Et_3_N.HCl. The crude precipitated product was filtered and washed thoroughly with water, then n-hexane and dried under vacuum. After purification on a Chromatotron (Centrifugal Thin-Layer Chromatograph) using n-hexane-ethyl acetate (2:1) as eluant and recrystallization from acetic acid yielded cycloadduct 3.

Light green needle crystals. (161 mg, 70%). [α]^21°C^_589_ = +12.0° (c= 0.01 g/ml, l=10 cm, acetone). *M*.p. 174–176°C. *R*_f_: 0.68 (ethyl acetate-n-hexane; 1:2). IR (KBr): ν = 3452, 3064, 2941 (C—H), 1708 (Cδb O),1599 (Cδb N), 1500, 1327, 1193, 1166, 1068, 844, 750, 696 cm^-1. 1^H NMR (400 MHz, CDCl_3_): δ= 8.17 (q, J=3.8 Hz 2H), 7.70 (t, J=4.7 Hz, 2H), 7.61 (t, J=7.6 Hz, 2H), 7.47 (t, J=6.5 Hz 2H), 7.28–7.41 (m, 5H), 7.05 (t, J=7.0, 1H), 5.44 (t, J=7.0 Hz 1H), 5.08–5.20 (dd, J=35.9 11.0 Hz 1H), 4.76–4.85 (dd, J=23.2 11.0 Hz 1H), 1.82 (t, J=7.3 Hz 3H). ^13^C NMR (100 MHz,CDCl_3_): δ=172.1(Cδb O), 171.4 (Cδb O),143.9 (Cδb N), 141.2, 138.6, 133.8, 130.9, 129.3, 128.6, 128.3, 127.6, 127.1, 125.5 (–CF_3_), 122.6, 121.9, 114.5, 65.3 (–CH), 52.9 (–CH), 51.6 (–CH), 16.4 (–CH_3_). GC—MS (70 eV): (m/*z*, %)= 463 (100) [*M*]^+^, 359 (80), 315 (30), 269 (10), 105 (40), 70 (43). Anal Calcd for C_26_H_20_F_3_N_3_O_2_ C, 67.38; H, 4.35; N, 9.07; found C, 66.45; H, 4.50; N, 8.74.

### Refinement

H atoms on C were placed in idealized positions with C—H distances 0.95 - 1.00 Å and there after treated as riding. A torsional parameter was refined for the methyl group. *U*_iso_ for H were assigned as 1.2 times *U*_eq_ of the attached atoms (1.5 for methyl).

### Crystal data

**Table:**

C_26_H_20_F_3_N_3_O_2_	*F*(000) = 960
*M**_r_* = 463.45	*D*_x_ = 1.446 Mg m^−^^3^
Orthorhombic, *P*2_1_2_1_2_1_	Cu *K*α radiation, λ = 1.54178 Å
Hall symbol: P 2ac 2ab	Cell parameters from 3807 reflections
*a* = 8.7982 (15) Å	θ = 3.4–65.8°
*b* = 9.3064 (15) Å	µ = 0.93 mm^−^^1^
*c* = 25.992 (4) Å	*T* = 90 K
*V* = 2128.2 (6) Å^3^	Lath, colourless
*Z* = 4	0.30 × 0.18 × 0.03 mm

### Data collection

**Table:**

Bruker Kappa APEXII CCD area-detector diffractometer	3822 independent reflections
Radiation source: fine-focus sealed tube	2959 reflections with *I* > 2σ(*I*)
graphite	*R*_int_ = 0.069
phi and ω scans	θ_max_ = 68.9°, θ_min_ = 3.4°
Absorption correction: multi-scan (*SADABS*; Sheldrick, 2004)	*h* = −10→10
*T*_min_ = 0.767, *T*_max_ = 0.972	*k* = −9→10
20719 measured reflections	*l* = −30→31

### Refinement

**Table:**

Refinement on *F*^2^	Hydrogen site location: inferred from neighbouring sites
Least-squares matrix: full	H-atom parameters constrained
*R*[*F*^2^ > 2σ(*F*^2^)] = 0.043	*w* = 1/[σ^2^(*F*_o_^2^) + (0.0512*P*)^2^ + 0.4052*P*] where *P* = (*F*_o_^2^ + 2*F*_c_^2^)/3
*wR*(*F*^2^) = 0.108	(Δ/σ)_max_ < 0.001
*S* = 1.03	Δρ_max_ = 0.31 e Å^−^^3^
3822 reflections	Δρ_min_ = −0.18 e Å^−^^3^
309 parameters	Extinction correction: *SHELXL97* (Sheldrick, 2008), Fc^*^=kFc[1+0.001xFc^2^λ^3^/sin(2θ)]^-1/4^
0 restraints	Extinction coefficient: 0.0034 (3)
Primary atom site location: structure-invariant direct methods	Absolute structure: Flack (1983), 1590 Friedel pairs
Secondary atom site location: difference Fourier map	Flack parameter: −0.2 (2)

### Special details

**Table:**

Geometry. All e.s.d.'s (except the e.s.d. in the dihedral angle between two l.s. planes) are estimated using the full covariance matrix. The cell e.s.d.'s are taken into account individually in the estimation of e.s.d.'s in distances, angles and torsion angles; correlations between e.s.d.'s in cell parameters are only used when they are defined by crystal symmetry. An approximate (isotropic) treatment of cell e.s.d.'s is used for estimating e.s.d.'s involving l.s. planes.
Refinement. Refinement of *F*^2^ against ALL reflections. The weighted *R*-factor *wR* and goodness of fit *S* are based on *F*^2^, conventional *R*-factors *R* are based on *F*, with *F* set to zero for negative *F*^2^. The threshold expression of *F*^2^ > σ(*F*^2^) is used only for calculating *R*-factors(gt) *etc*. and is not relevant to the choice of reflections for refinement. *R*-factors based on *F*^2^ are statistically about twice as large as those based on *F*, and *R*- factors based on ALL data will be even larger.

### Fractional atomic coordinates and isotropic or equivalent isotropic displacement parameters (Å^2^)

**Table:**

	*x*	*y*	*z*	*U*_iso_*/*U*_eq_	
F1	0.2050 (3)	−0.3740 (2)	0.74363 (8)	0.0649 (7)	
F2	0.0123 (2)	−0.2462 (3)	0.76478 (7)	0.0556 (6)	
F3	0.2228 (2)	−0.2229 (2)	0.80549 (7)	0.0451 (5)	
O1	0.4288 (2)	0.4051 (2)	0.67014 (8)	0.0321 (5)	
O2	0.5158 (2)	0.4981 (2)	0.49986 (8)	0.0312 (5)	
N1	0.4518 (3)	0.4819 (2)	0.58594 (9)	0.0251 (5)	
N2	0.4312 (3)	0.1795 (2)	0.51974 (9)	0.0254 (6)	
N3	0.3502 (3)	0.0970 (2)	0.55431 (9)	0.0242 (5)	
C1	0.4613 (3)	0.3818 (3)	0.62588 (12)	0.0278 (6)	
C2	0.5047 (3)	0.4301 (3)	0.53947 (12)	0.0278 (7)	
C3	0.5441 (3)	0.2709 (3)	0.54625 (11)	0.0252 (6)	
H3	0.6507	0.2485	0.5357	0.030*	
C4	0.5150 (3)	0.2394 (3)	0.60326 (11)	0.0270 (6)	
H4	0.6076	0.2018	0.6210	0.032*	
C5	0.3890 (3)	0.1289 (3)	0.60086 (11)	0.0253 (6)	
C6	0.4527 (3)	0.1235 (3)	0.46979 (11)	0.0239 (6)	
C7	0.3790 (3)	−0.0028 (3)	0.45435 (11)	0.0265 (6)	
H7	0.3155	−0.0533	0.4777	0.032*	
C8	0.3995 (3)	−0.0536 (3)	0.40461 (11)	0.0284 (7)	
H8	0.3496	−0.1392	0.3941	0.034*	
C9	0.4919 (3)	0.0190 (3)	0.37010 (12)	0.0315 (7)	
H9	0.5066	−0.0172	0.3363	0.038*	
C10	0.5626 (4)	0.1450 (3)	0.38552 (11)	0.0310 (7)	
H10	0.6254	0.1954	0.3619	0.037*	
C11	0.5429 (3)	0.1988 (3)	0.43503 (11)	0.0281 (7)	
H11	0.5906	0.2860	0.4450	0.034*	
C12	0.3202 (4)	0.0518 (3)	0.64432 (11)	0.0274 (7)	
C13	0.3844 (4)	0.0538 (3)	0.69304 (11)	0.0312 (7)	
H13	0.4651	0.1185	0.7003	0.037*	
C14	0.3318 (4)	−0.0378 (3)	0.73130 (11)	0.0321 (7)	
H14	0.3778	−0.0376	0.7644	0.039*	
C15	0.2117 (4)	−0.1294 (3)	0.72079 (11)	0.0301 (7)	
C16	0.1408 (3)	−0.1260 (3)	0.67349 (11)	0.0302 (7)	
H16	0.0559	−0.1865	0.6671	0.036*	
C17	0.1931 (3)	−0.0344 (3)	0.63522 (11)	0.0279 (7)	
H17	0.1425	−0.0304	0.6030	0.033*	
C18	0.3916 (3)	0.6287 (3)	0.59575 (12)	0.0277 (7)	
H18	0.3286	0.6223	0.6277	0.033*	
C19	0.5216 (3)	0.7315 (3)	0.60757 (11)	0.0260 (6)	
C20	0.5713 (3)	0.7462 (3)	0.65816 (12)	0.0310 (7)	
H20	0.5256	0.6896	0.6843	0.037*	
C21	0.6860 (4)	0.8416 (3)	0.67111 (13)	0.0355 (8)	
H21	0.7179	0.8506	0.7059	0.043*	
C22	0.7537 (3)	0.9240 (3)	0.63311 (13)	0.0361 (8)	
H22	0.8316	0.9904	0.6418	0.043*	
C23	0.7077 (4)	0.9093 (3)	0.58217 (13)	0.0338 (7)	
H23	0.7553	0.9648	0.5561	0.041*	
C24	0.5922 (3)	0.8135 (3)	0.56927 (12)	0.0280 (7)	
H24	0.5613	0.8039	0.5344	0.034*	
C25	0.2853 (4)	0.6748 (3)	0.55281 (12)	0.0313 (7)	
H25A	0.3429	0.6852	0.5208	0.047*	
H25B	0.2383	0.7670	0.5617	0.047*	
H25C	0.2059	0.6021	0.5482	0.047*	
C26	0.1630 (4)	−0.2415 (4)	0.75849 (12)	0.0402 (8)	

### Atomic displacement parameters (Å^2^)

**Table:**

	*U*^11^	*U*^22^	*U*^33^	*U*^12^	*U*^13^	*U*^23^
F1	0.110 (2)	0.0300 (11)	0.0548 (13)	0.0067 (13)	0.0139 (13)	0.0070 (10)
F2	0.0414 (13)	0.0789 (16)	0.0464 (12)	−0.0149 (12)	−0.0022 (9)	0.0186 (11)
F3	0.0419 (12)	0.0597 (13)	0.0336 (10)	0.0020 (10)	−0.0040 (8)	0.0095 (9)
O1	0.0368 (13)	0.0260 (12)	0.0335 (12)	0.0022 (10)	−0.0047 (10)	−0.0027 (9)
O2	0.0277 (11)	0.0264 (11)	0.0394 (12)	−0.0006 (9)	0.0035 (9)	0.0002 (9)
N1	0.0242 (13)	0.0189 (13)	0.0322 (13)	0.0008 (10)	−0.0027 (10)	−0.0019 (10)
N2	0.0193 (12)	0.0227 (13)	0.0343 (13)	−0.0037 (10)	0.0008 (10)	−0.0049 (10)
N3	0.0186 (13)	0.0184 (13)	0.0356 (13)	0.0004 (10)	0.0021 (10)	0.0014 (10)
C1	0.0204 (15)	0.0238 (16)	0.0391 (17)	0.0026 (13)	−0.0037 (12)	−0.0027 (13)
C2	0.0189 (16)	0.0257 (16)	0.0387 (17)	−0.0009 (13)	−0.0013 (13)	−0.0058 (13)
C3	0.0156 (14)	0.0202 (15)	0.0397 (16)	−0.0008 (12)	−0.0007 (12)	−0.0054 (12)
C4	0.0232 (16)	0.0215 (15)	0.0361 (15)	0.0047 (13)	−0.0050 (12)	−0.0026 (13)
C5	0.0184 (15)	0.0230 (15)	0.0343 (16)	0.0052 (12)	−0.0034 (12)	−0.0021 (13)
C6	0.0173 (14)	0.0202 (15)	0.0342 (15)	0.0008 (12)	0.0000 (11)	−0.0029 (12)
C7	0.0193 (15)	0.0217 (15)	0.0385 (17)	−0.0025 (13)	0.0023 (12)	0.0005 (13)
C8	0.0256 (17)	0.0212 (16)	0.0384 (17)	−0.0020 (12)	−0.0007 (13)	−0.0044 (12)
C9	0.0281 (17)	0.0299 (18)	0.0366 (16)	−0.0020 (14)	0.0015 (13)	−0.0022 (13)
C10	0.0267 (17)	0.0288 (17)	0.0376 (17)	−0.0027 (14)	0.0029 (13)	0.0025 (13)
C11	0.0215 (15)	0.0234 (16)	0.0394 (17)	−0.0035 (13)	0.0006 (13)	−0.0021 (12)
C12	0.0298 (17)	0.0199 (15)	0.0324 (16)	0.0069 (12)	−0.0015 (13)	0.0002 (12)
C13	0.0306 (17)	0.0242 (17)	0.0388 (17)	0.0004 (14)	−0.0054 (14)	−0.0065 (13)
C14	0.0349 (19)	0.0285 (17)	0.0329 (17)	0.0058 (14)	−0.0038 (14)	−0.0015 (13)
C15	0.0307 (18)	0.0284 (17)	0.0311 (15)	0.0043 (15)	−0.0005 (13)	0.0027 (14)
C16	0.0245 (16)	0.0279 (17)	0.0382 (17)	0.0013 (14)	0.0028 (13)	−0.0002 (14)
C17	0.0251 (16)	0.0282 (17)	0.0304 (16)	0.0037 (13)	0.0003 (13)	0.0002 (13)
C18	0.0256 (16)	0.0201 (15)	0.0373 (16)	0.0053 (13)	−0.0006 (13)	−0.0041 (13)
C19	0.0212 (15)	0.0159 (14)	0.0408 (16)	0.0056 (12)	−0.0040 (12)	−0.0036 (12)
C20	0.0296 (17)	0.0255 (16)	0.0380 (17)	0.0054 (14)	−0.0006 (13)	−0.0045 (13)
C21	0.0290 (18)	0.0323 (19)	0.0452 (19)	0.0071 (15)	−0.0062 (15)	−0.0105 (15)
C22	0.0233 (17)	0.0250 (17)	0.060 (2)	−0.0008 (14)	−0.0023 (15)	−0.0122 (15)
C23	0.0255 (17)	0.0242 (17)	0.052 (2)	0.0040 (14)	0.0048 (14)	−0.0017 (14)
C24	0.0233 (16)	0.0233 (16)	0.0373 (17)	0.0049 (13)	−0.0017 (13)	−0.0023 (12)
C25	0.0278 (17)	0.0238 (16)	0.0424 (18)	0.0029 (14)	−0.0029 (14)	−0.0014 (13)
C26	0.045 (2)	0.042 (2)	0.0335 (17)	−0.0040 (17)	−0.0003 (15)	0.0002 (16)

### Geometric parameters (Å, °)

**Table:**

F1—C26	1.344 (4)	C11—H11	0.9500
F2—C26	1.337 (4)	C12—C13	1.387 (4)
F3—C26	1.342 (4)	C12—C17	1.396 (4)
O1—C1	1.205 (3)	C13—C14	1.390 (4)
O2—C2	1.213 (4)	C13—H13	0.9500
N1—C2	1.381 (4)	C14—C15	1.385 (5)
N1—C1	1.397 (4)	C14—H14	0.9500
N1—C18	1.487 (4)	C15—C16	1.379 (4)
N2—N3	1.381 (3)	C15—C26	1.494 (4)
N2—C6	1.412 (4)	C16—C17	1.389 (4)
N2—C3	1.477 (4)	C16—H16	0.9500
N3—C5	1.292 (4)	C17—H17	0.9500
C1—C4	1.525 (4)	C18—C25	1.519 (4)
C2—C3	1.532 (4)	C18—C19	1.523 (4)
C3—C4	1.532 (4)	C18—H18	1.0000
C3—H3	1.0000	C19—C20	1.393 (4)
C4—C5	1.514 (4)	C19—C24	1.400 (4)
C4—H4	1.0000	C20—C21	1.386 (4)
C5—C12	1.469 (4)	C20—H20	0.9500
C6—C11	1.392 (4)	C21—C22	1.385 (4)
C6—C7	1.401 (4)	C21—H21	0.9500
C7—C8	1.388 (4)	C22—C23	1.391 (4)
C7—H7	0.9500	C22—H22	0.9500
C8—C9	1.386 (4)	C23—C24	1.392 (4)
C8—H8	0.9500	C23—H23	0.9500
C9—C10	1.386 (4)	C24—H24	0.9500
C9—H9	0.9500	C25—H25A	0.9800
C10—C11	1.391 (4)	C25—H25B	0.9800
C10—H10	0.9500	C25—H25C	0.9800
			
C2—N1—C1	113.4 (2)	C12—C13—H13	119.7
C2—N1—C18	126.2 (2)	C14—C13—H13	119.7
C1—N1—C18	120.4 (2)	C15—C14—C13	119.4 (3)
N3—N2—C6	117.5 (2)	C15—C14—H14	120.3
N3—N2—C3	111.3 (2)	C13—C14—H14	120.3
C6—N2—C3	123.5 (2)	C16—C15—C14	120.4 (3)
C5—N3—N2	110.2 (2)	C16—C15—C26	118.1 (3)
O1—C1—N1	125.1 (3)	C14—C15—C26	121.3 (3)
O1—C1—C4	126.7 (3)	C15—C16—C17	120.2 (3)
N1—C1—C4	108.1 (2)	C15—C16—H16	119.9
O2—C2—N1	126.0 (3)	C17—C16—H16	119.9
O2—C2—C3	125.8 (3)	C16—C17—C12	119.8 (3)
N1—C2—C3	108.3 (3)	C16—C17—H17	120.1
N2—C3—C2	110.5 (2)	C12—C17—H17	120.1
N2—C3—C4	103.2 (2)	N1—C18—C25	110.7 (2)
C2—C3—C4	105.0 (2)	N1—C18—C19	110.2 (2)
N2—C3—H3	112.5	C25—C18—C19	115.7 (2)
C2—C3—H3	112.5	N1—C18—H18	106.6
C4—C3—H3	112.5	C25—C18—H18	106.6
C5—C4—C1	112.3 (2)	C19—C18—H18	106.6
C5—C4—C3	102.2 (2)	C20—C19—C24	118.6 (3)
C1—C4—C3	105.0 (2)	C20—C19—C18	119.2 (3)
C5—C4—H4	112.2	C24—C19—C18	122.2 (3)
C1—C4—H4	112.2	C21—C20—C19	121.4 (3)
C3—C4—H4	112.2	C21—C20—H20	119.3
N3—C5—C12	119.9 (3)	C19—C20—H20	119.3
N3—C5—C4	112.9 (3)	C22—C21—C20	119.7 (3)
C12—C5—C4	127.0 (3)	C22—C21—H21	120.2
C11—C6—C7	120.0 (3)	C20—C21—H21	120.2
C11—C6—N2	119.2 (3)	C21—C22—C23	119.9 (3)
C7—C6—N2	120.8 (3)	C21—C22—H22	120.0
C8—C7—C6	119.5 (3)	C23—C22—H22	120.0
C8—C7—H7	120.2	C22—C23—C24	120.3 (3)
C6—C7—H7	120.2	C22—C23—H23	119.9
C9—C8—C7	120.8 (3)	C24—C23—H23	119.9
C9—C8—H8	119.6	C23—C24—C19	120.1 (3)
C7—C8—H8	119.6	C23—C24—H24	119.9
C8—C9—C10	119.2 (3)	C19—C24—H24	119.9
C8—C9—H9	120.4	C18—C25—H25A	109.5
C10—C9—H9	120.4	C18—C25—H25B	109.5
C9—C10—C11	121.1 (3)	H25A—C25—H25B	109.5
C9—C10—H10	119.5	C18—C25—H25C	109.5
C11—C10—H10	119.5	H25A—C25—H25C	109.5
C10—C11—C6	119.3 (3)	H25B—C25—H25C	109.5
C10—C11—H11	120.3	F2—C26—F3	106.4 (3)
C6—C11—H11	120.3	F2—C26—F1	106.1 (3)
C13—C12—C17	119.3 (3)	F3—C26—F1	105.8 (3)
C13—C12—C5	121.8 (3)	F2—C26—C15	112.8 (3)
C17—C12—C5	118.7 (3)	F3—C26—C15	113.2 (3)
C12—C13—C14	120.6 (3)	F1—C26—C15	111.9 (3)
			
C6—N2—N3—C5	154.5 (3)	C8—C9—C10—C11	0.4 (5)
C3—N2—N3—C5	3.9 (3)	C9—C10—C11—C6	1.0 (5)
C2—N1—C1—O1	176.3 (3)	C7—C6—C11—C10	−1.9 (4)
C18—N1—C1—O1	−2.8 (4)	N2—C6—C11—C10	−179.1 (3)
C2—N1—C1—C4	−5.3 (3)	N3—C5—C12—C13	162.7 (3)
C18—N1—C1—C4	175.6 (2)	C4—C5—C12—C13	−12.0 (5)
C1—N1—C2—O2	−175.9 (3)	N3—C5—C12—C17	−12.5 (4)
C18—N1—C2—O2	3.1 (5)	C4—C5—C12—C17	172.8 (3)
C1—N1—C2—C3	5.2 (3)	C17—C12—C13—C14	5.6 (4)
C18—N1—C2—C3	−175.8 (3)	C5—C12—C13—C14	−169.6 (3)
N3—N2—C3—C2	−115.0 (3)	C12—C13—C14—C15	−1.5 (5)
C6—N2—C3—C2	96.5 (3)	C13—C14—C15—C16	−2.5 (5)
N3—N2—C3—C4	−3.1 (3)	C13—C14—C15—C26	172.8 (3)
C6—N2—C3—C4	−151.7 (2)	C14—C15—C16—C17	2.5 (5)
O2—C2—C3—N2	−71.1 (4)	C26—C15—C16—C17	−173.0 (3)
N1—C2—C3—N2	107.8 (3)	C15—C16—C17—C12	1.6 (4)
O2—C2—C3—C4	178.2 (3)	C13—C12—C17—C16	−5.6 (4)
N1—C2—C3—C4	−2.9 (3)	C5—C12—C17—C16	169.7 (3)
O1—C1—C4—C5	71.2 (4)	C2—N1—C18—C25	43.8 (4)
N1—C1—C4—C5	−107.2 (3)	C1—N1—C18—C25	−137.3 (3)
O1—C1—C4—C3	−178.5 (3)	C2—N1—C18—C19	−85.4 (3)
N1—C1—C4—C3	3.1 (3)	C1—N1—C18—C19	93.5 (3)
N2—C3—C4—C5	1.4 (3)	N1—C18—C19—C20	−88.0 (3)
C2—C3—C4—C5	117.2 (2)	C25—C18—C19—C20	145.5 (3)
N2—C3—C4—C1	−116.0 (2)	N1—C18—C19—C24	92.7 (3)
C2—C3—C4—C1	−0.2 (3)	C25—C18—C19—C24	−33.8 (4)
N2—N3—C5—C12	−178.4 (2)	C24—C19—C20—C21	1.3 (4)
N2—N3—C5—C4	−2.9 (3)	C18—C19—C20—C21	−178.0 (3)
C1—C4—C5—N3	112.9 (3)	C19—C20—C21—C22	−0.4 (4)
C3—C4—C5—N3	0.9 (3)	C20—C21—C22—C23	−0.7 (5)
C1—C4—C5—C12	−72.1 (4)	C21—C22—C23—C24	0.9 (5)
C3—C4—C5—C12	175.9 (3)	C22—C23—C24—C19	0.1 (4)
N3—N2—C6—C11	−175.5 (3)	C20—C19—C24—C23	−1.2 (4)
C3—N2—C6—C11	−28.7 (4)	C18—C19—C24—C23	178.1 (3)
N3—N2—C6—C7	7.4 (4)	C16—C15—C26—F2	−50.1 (4)
C3—N2—C6—C7	154.2 (3)	C14—C15—C26—F2	134.5 (3)
C11—C6—C7—C8	1.4 (4)	C16—C15—C26—F3	−171.0 (3)
N2—C6—C7—C8	178.5 (3)	C14—C15—C26—F3	13.6 (5)
C6—C7—C8—C9	0.0 (4)	C16—C15—C26—F1	69.5 (4)
C7—C8—C9—C10	−0.9 (5)	C14—C15—C26—F1	−105.9 (4)

### Hydrogen-bond geometry (Å, °)

**Table:**

*D*—H···*A*	*D*—H	H···*A*	*D*···*A*	*D*—H···*A*
C18—H18···O1	1.00	2.47	2.859 (4)	103
C8—H8···Cg1^i^	0.95	2.58	3.491 (3)	161

Symmetry codes: (i) −*x*−1, *y*+1/2, −*z*+3/2.
